# Caveolin-1 expression predicts efficacy of weekly nab-paclitaxel plus gemcitabine for metastatic breast cancer in the phase II clinical trial

**DOI:** 10.1186/s12885-018-4936-y

**Published:** 2018-10-22

**Authors:** Yannan Zhao, Fangfang Lv, She Chen, Zhonghua Wang, Jian Zhang, Sheng Zhang, Jun Cao, Leiping Wang, Enying Cao, Biyun Wang, Xichun Hu

**Affiliations:** 1 Department of Medical Oncology, Fudan University Shanghai Cancer Center, 270 Dong an road, 200032 Shanghai, People’s Republic of China; 2 Department of Biochemistry and Molecular Biology, Shanghai Medical College, 130 Dong an road, 200032 Shanghai, People’s Republic of China

**Keywords:** Metastatic breast cancer, Chemotherapy, Nab-paclitaxel, Gemcitabine, Caveolin-1

## Abstract

**Background:**

Nanoparticle albumin-bound (nab)-paclitaxel has better efficacy, safety profiles, and no need to use prophylactic steroids compared with solvent-based paclitaxel. We performed a single arm, phase II study to evaluate the efficacy and safety of weekly nab-paclitaxel and gemcitabine combination in patients with metastatic breast cancer (MBC) and explored role of tumor/stromal Caveolin-1 (Cav-1) as a predictive biomarker for the efficacy.

**Methods:**

Nab-paclitaxel (125 mg/m^2^) and gemcitabine (800 mg/m^2^) were administered on days 1, 8, and 15 in a 4-week cycle. The primary end point was objective response rate (ORR). Secondary end points were progression free survival (PFS), overall survival (OS) and safety profile. Exploratory study included immunohistochemical detection of Cav-1.

**Results:**

Among 85 patients enrolled in the study, ORR was 52.4%. After a median follow-up of 17.2 months, median PFS was 7.9 months (95%CI, 6.6–9.2) and median OS was 25.8 months (95% CI, 20.4–31.1). The most common toxicities were neutropenia (75.0% for all grades; 45.2% for grade 3 or worse) and the most common non-hematologic toxicity was peripheral neuropathy (50.0% for all grades, 7.14% for grade 3 or worse). Higher tumor Cav-1 level and lower stromal Cav-1 level were significantly associated with longer PFS of nab-paclitaxel and gemcitabine.

**Conclusions:**

The regimen had substantial antitumor activity and was well tolerated in MBC patients. Tumor/stromal Cav-1 level may be a good predictor for the efficacy of nab-paclitaxel and gemcitabine.

**Trial registration:**

NCT01550848. Registered 12 March 2012.

**Electronic supplementary material:**

The online version of this article (10.1186/s12885-018-4936-y) contains supplementary material, which is available to authorized users.

## Background

Metastatic breast cancer (MBC) is known as an incurable disease with a median 5-year survival rate around 23.8–30% [1]. Taxane is considered to be one of the most effective treatments for both early breast cancer and MBC [2].

Abraxane, a CrEL-free, protein-stabilized, nanoparticle albumin-bound paclitaxel (nab-paclitaxel), proved to be an effective agent for MBC treatment [3]. It has an albumin delivery system that increases drug targeting to the tumor cell and can be safely infused in 30 min at a high dose of 260 mg/m^2^ every 3 weeks without premedication [4].

Combination of gemcitabine and paclitaxel is a conventional cytotoxic chemotherapy doublet and has been recommended in National Comprehensive Cancer Network (NCCN) guideline as one of the effective combinations in MBC patients pretreated with anthracycline [5]. Therefore, combination of nab-paclitaxel and gemcitabine has been exploring as a more effective regimen in MBC setting. Roy et al. [6] conducted a single-arm phase II trial in US to evaluate weekly nab-paclitaxel and gemcitabine combination in MBC patients as first-line treatment. It revealed ORR of 50% and median PFS of 7.9 months in 50 MBC patients. In another phase II trial conducted in Europe [7], nab-paclitaxel (150 mg/m^2^), gemcitabine (1500 mg/m^2^) and bevacizumab (10 mg/kg) were administered to TNBC patients as first-line therapy on day 1 and 15 of a 28-day cycle, with ORR of 75.9% and mPFS of 10.4 months. Accordingly, the combination of weekly nab-paclitaxel and gemcitabine appears to be an effective combination therapy for MBC patients in the first-line setting.

The prominent way of nab-paclitaxel transport appears to be through receptor-mediated transcytosis, which is mediated by Caveolin-1 (Cav-1) [8]. Cav-1 is the main part of caveolae (flask-shaped invaginations of plasma membrane) and facilitates transport of albumin through transcytosis pathway. Dysregulation of tumor Cav-1 plays an important role in tumorigenesis of breast cancer [9]. In addition, loss of stromal Cav-1 is associated with early disease recurrence, poorer progression free survival, and tamoxifen-resistance in breast cancer patients [10]. Based on these facts, we suppose that tumor and stromal Cav-1 expression may be a predictor of nab-paclitaxel efficacy.

Considering these studies, we initiated this phase II study to assess the efficacy and safety profiles of weekly nab-paclitaxel with gemcitabine in a 4-week regimen setting among MBC patients. We also evaluated that the association between the tumor cell/stromal expression of Cav-1 in breast cancer patients and the efficacy of nab-paclitaxel and gemcitabine.

## Methods

### Patients

Women between 18 and 70 years of age, with histologically confirmed advanced or MBC, were eligible to participate in the study. Patients with luminal, HER2-positive or triple-negative breast cancer were allowed to enroll in this study. Luminal breast cancer was histologically confirmed estrogen or progesterone receptors positive (tumor tissue that expressed estrogen receptor (ER)/ progesterone receptor (PR) in at ≥10% of the cells). HER2 positive was defined as either fluorescence in situ hybridization positive, 3+ staining intensity by immunohistochemistry, or 2+ by immunohistochemistry and fluorescence in situ hybridization positive. Triple-negative breast cancer is pathologically defined as ER negative, PR negative and HER2-negative disease. Patients were required to have Eastern Cooperative Oncology Group (ECOG) performance status of 0–1; evidence of adequate organ function; and a life expectancy for at least 3 months. Patients were also required to have measurable disease according to the Response Evaluation Criteria in Solid Tumors (RECIST) 1.1. Patients who underwent taxane therapy as a part of adjuvant or neoadjuvant breast cancer treatment were required to have an interval for at least 12 months before the study. Patients who received prior taxanes in the metastatic setting were required an interval for at least 3 months before the enrollment. The exclusion criteria were the following: 1) patients with clinical evidence of brain metastases or serious concurrent diseases; 2) patients with pre-existing grade 1 peripheral neuropathy or worse; 3) patients who underwent combined hormonal therapy or immunotherapy; 4) patients with other malignancies within last 5 years, which could affect the diagnosis or assessment of breast cancer; 5) patients who underwent radiotherapy, chemotherapy or administration of any other drug that is being investigated within 4 weeks prior to enrollment; 6) pregnancy.

The trial was conducted in accordance with the principles of Good Clinical Practice and the Declaration of Helsinki. Study procedures were approved by the Ethics Committee and Institutional Review Board of Fudan University Shanghai Cancer Center on Nov 28, 2011. We confirmed that all ongoing and related trials for these drugs were registered. All patients provided written informed consent prior to any study-related procedures.

### Study design and treatment

This was a single-arm phase II study. Eligible patients received weekly treatment of nab-paclitaxel and gemcitabine combination, every 4 weeks. Patients received nab-paclitaxel and gemcitabine intravenously at a dose of 125 mg/m2 and 800 mg/m2, over 30 min, on days 1, 8, and 15, respectively, without corticosteroid or antihistamine premedication or special infusion sets. All HER2-positive patients received trastuzumab concurrently. Treatment was continued until disease progression, intolerable toxicities, or consent withdrawal.

Tumor assessment was performed at baseline, and then every two cycles until disease progression, according to the RECIST 1.1. It was performed using computed tomography, spiral computed tomography, or magnetic resonance imaging. Safety was assessed at each cycle; adverse events were graded using the National Cancer Institute Common Terminology Criteria for Adverse Events (NCI-CTCAE) version 4.0. Data of adverse events were collected up to 28 days after administration of the last dose. After the treatment period, the patients were followed-up every 3 months for one year after the last patient was recruited.

The primary end point was objective response rate (ORR). Secondary end points were progression free survival (PFS), overall survival (OS) and safety profile. ORR was defined as the percentage of evaluable patients with measurable disease at baseline who had the best objective tumour response of complete response (CR) or partial response (PR). PFS was defined as the time between the date of enrollment and the date of the earliest evidence of objective disease progression or death from any cause before documented disease progression. OS was defined as the time interval between enrollment and death in follow-ups. Safety profile was used to evaluate frequency and severity of all adverse events in patients who received the regimens.

### Exploratory analysis

For immunohistochemistry, formalin fixed paraffin-embedded (FFPE) tissue was requested from all enrolled patients, and 45 patients were available for FFPE tissue. 5-μm thick slides were deparaffinised in xylene (3 changes of 2 min each) and then rehydrated through graduated alcohols of 2 min each (100%, 95%, and 70%) and ended with distilled water. The slides were placed in a 3% peroxidase block for 5 min. Heat induced antigen retrieval was done for 30 min in a microwave oven in 10 mM, pH 6.0 citrate buffer. Then, the slides were incubated with the primary antibodies for Cav-1 (rabbit polyclonal antibody, Proteintech, 16,447–1-AP, Wuhan, China) at a dilution 1:200. The antigen-antibody reaction was visualized by Thermo scientific UltraVision LP Detection System. After primary antibody incubation the slides were rinsed and incubated with a horseradish peroxidase polymer 2-step system conjugated anti-rabbit for 20 min. Slides were rinsed again in Tris buffered saline, incubated with DAB+ (Dako) for 5 min, rinsed, and counterstained with hematoxylin for 10 s. Slides were then rinsed in ammonia water and dehydrated following the opposite order (70%, 95%, and 100% alcohol) that ended in xylene, then mounted, and cover slipped.

Cav-1 expression was assessed in tumor tissue for staining intensity (0–3) and percentage of cells staining positive (0 for 0%, 1 for ≤10%, 2 for 11–50%, 3 for 51–75%, 4 for ≥75%) which was used to derive an H-score ranging from 0 to 12, and in stroma tissue for staining intensity (0–3). The cut-off point was the median value of staining score.

### Statistical analysis

We hypothesized an ORR rate of 55% with the addition of gemcitabine in the whole population, compared to 40% for weekly nab-paclitaxel monotherapy, as reported by previous studies. At least 38 responding patients in a total of 76 patients were required to reach 80% power to detect a statistically significant difference (type 1 error α = 0.05) in ORR between the treatment group and historical control. Consequently, 84 patients were required with 10% drop-out rate.

All statistical analyses were based on Full Analysis Set (FAS) population, which included all subjects who did not fail to satisfy major entry criteria (irrespective of whether they were treated or not). ORR and baseline characteristics were compared between subgroups using Wald χ2 tests. OS and PFS were analyzed using the Kaplan-Meier method and were compared between subgroups using the Log-rank test. Multivariate analyses with Cox proportional hazards regression models were used to identify independent prognostic factors of PFS in all patients. For the exploratory analysis, patients were divided into 2 groups based on tumor/stromal expression levels: for tumor H-score, high (median or greater) versus low (less than median); for stroma intensity score: high (median or greater) versus low (less than median). All statistical analyses were carried out using SPSS 20.0 software (IBM Corporation, Armonk, NY, USA). The statistical difference was considered significant if the *p* value was less than 0.05 (ClinicalTrials.gov identifier: NCT01550848).

## Results

### Patients

**Fig. 1:**
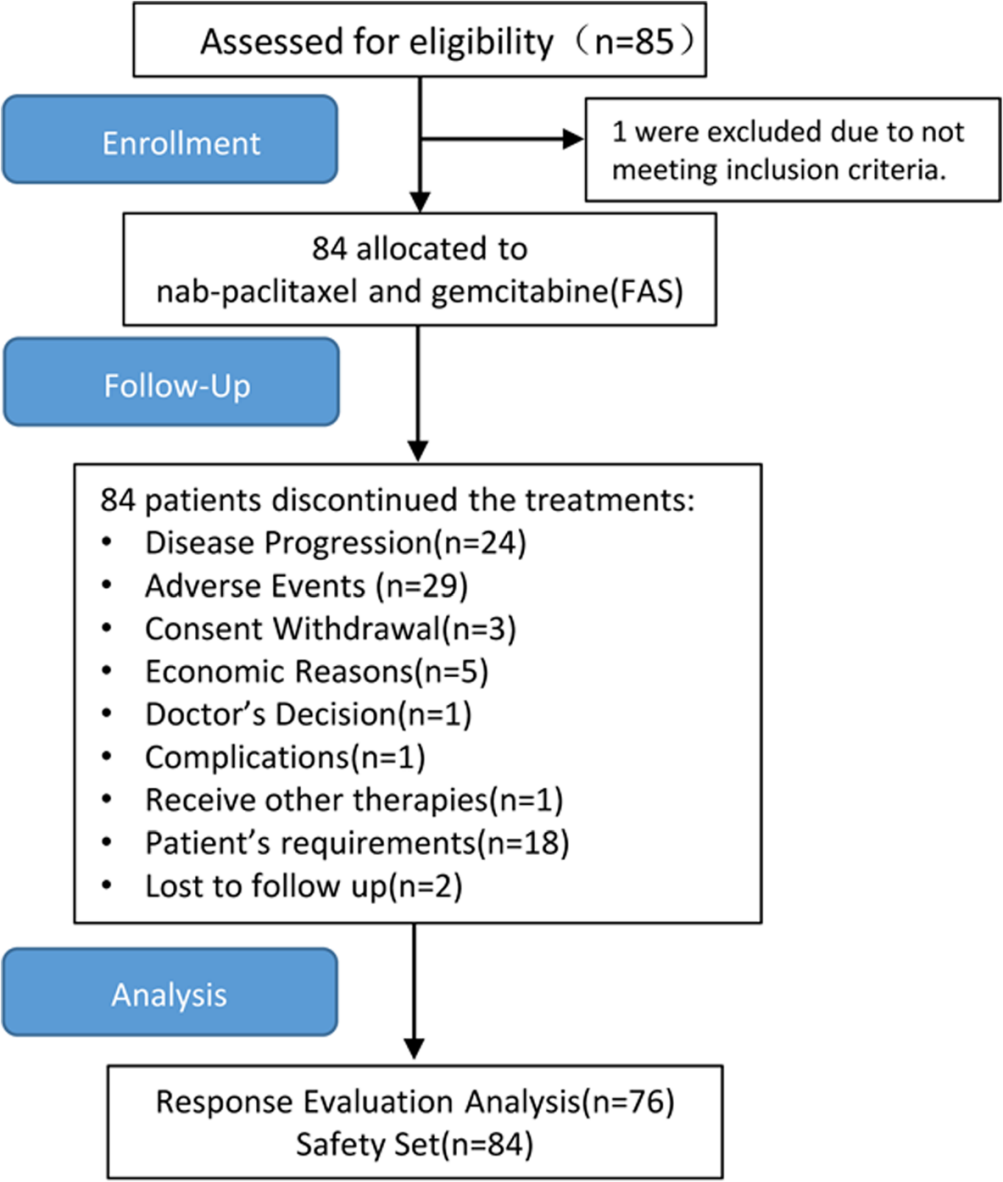
Flowchart of phase II trial of nab-paclitaxel and gemcitabine

A total of 85 women, who signed the informed consent form from Jan 2012 to Jul 2014 were enrolled in the study. 84 of them met the eligibility criteria and formed the FAS population. All eligible patients received at least one dose of nab-paclitaxel and gemcitabine, and were included in the safety analyses (Fig. 1). Demographic and baseline characteristics of the FAS population are listed in Table 1. The median age of patients was 50.5 years (range, 28–70 years); 61 patients (72.6%) were classified as luminal subtype; 59 patients (70.2%) received nab-paclitaxel and gemcitabine as first-line chemotherapy; 55 patients (65.5%) underwent prior taxane therapy; while 69 patients (82.1%) had visceral metastases.

**Table 1:** Patient characteristics (*n* = 84)

Characteristic	Whole population (*n* = 84) Number (%)
Median age, years (range)	50.5(28–70)
Amenorrhea	
Premenopausal	36 (42.9)
Postmenopausal	48 (57.1)
Advanced or metastatic	
De novo metastatic	12 (14.3)
Metastatic	72 (85.7)
No. of metastatic sites	
1	6 (7.1)
2	31 (36.9)
≥ 3	47 (56.0)
Metastatic sites	
Visceral	69 (82.1)
Lung	39 (46.4)
Liver	48 (57.1)
Non-visceral	15 (17.9)
Bone	46 (54.8)
Subgroup	
Luminal	61 (72.6)
Triple-negative	12 (14.3)
HER2 positive	10(11.9)
Unknown	1(1.2)
Lines of chemotherapy	
First line	59(70.2)
Second line or more line	25(29.7)
Prior chemotherapy	
Adjuvant/neoadjuvant (*n* = 65)	
Anthracycline-containing	63 (96.9)
Taxane-containing	49 (75.4)
Both	47 (72.3)
Chemotherapy for MBC (*n* = 25)	
Anthracycline-containing	6 (24)
Taxane-containing	10 (40)
Both	3(12)

### Efficacy

At cutoff date of the data collection (Jun, 2015), 75 patients (89.3%) experienced a disease progression, while 38 patients (45.2%) died. Two patients (2.4%) achieved CR, while forty-two (50.0%) achieved PR, accounting for an ORR of 52.4%. Twenty-two patients (26.2%) had stable disease (SD), and ten (11.9%) had progression disease (PD). Additionally, eight patients were not evaluable. Among those, 3 patients withdrew the consent due to economic factors; 2 patients discontinued due to no recovery of grade 3 peripheral neuropathy after a 2 weeks’ delay; 1 patient discontinued due to grade 2 skin toxicity; 1 patient was lost to follow-up visit; 1 patient was evaluated as ECOG 2 at the beginning of cycle 2, and consequent chemotherapy was stopped. Among the 44 patients who responded, most patients (33/44, 75.0%) achieved the best response during second cycle.

**Fig. 2:**
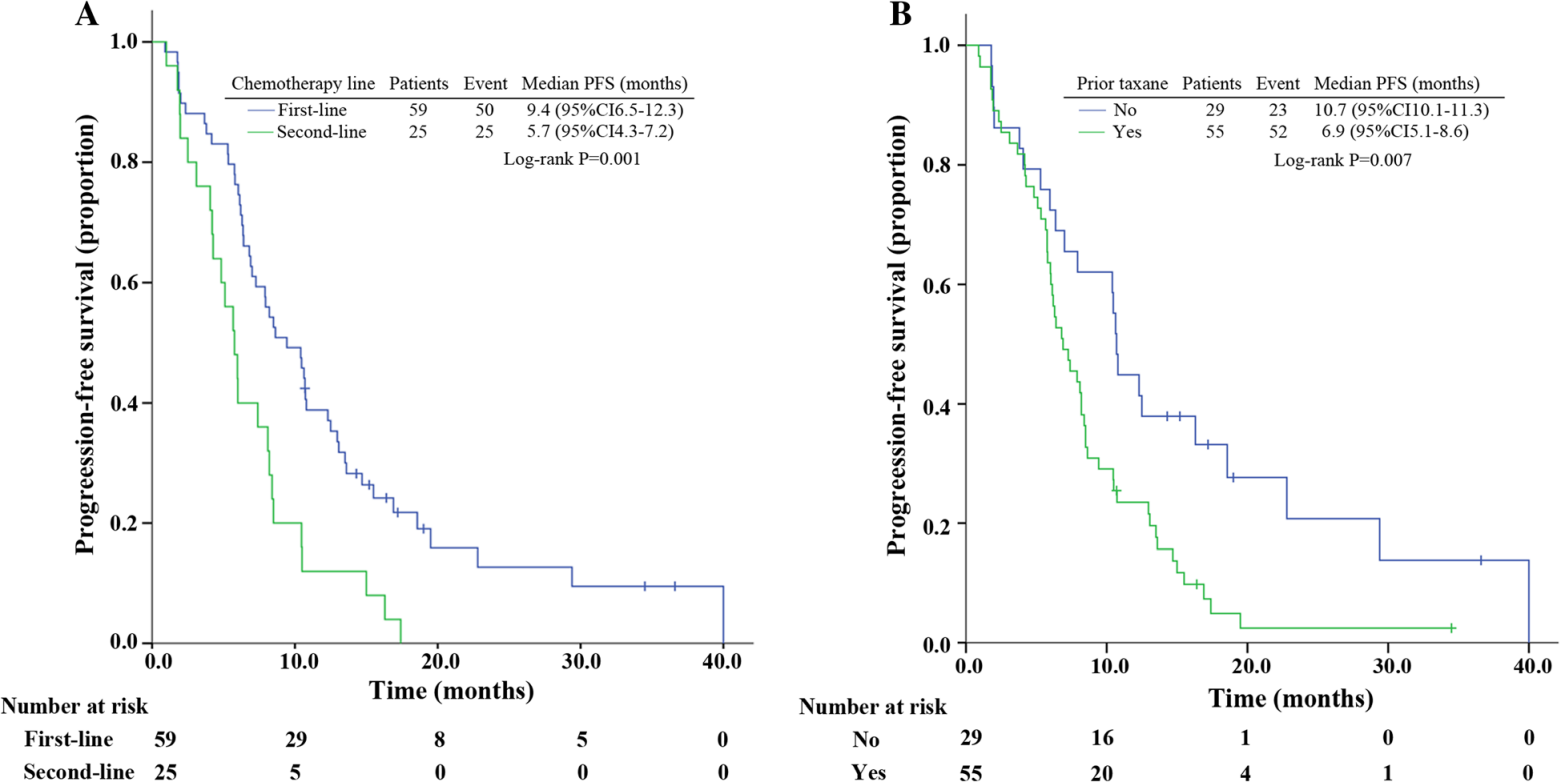
Kaplan–Meier curves for progression-free survival. **a** For patients stratified by lines of therapy. **b** For patients stratified by prior taxane. Abbreviations: CI, confidence interval; PFS, progression-free survival

**Fig. 3:**
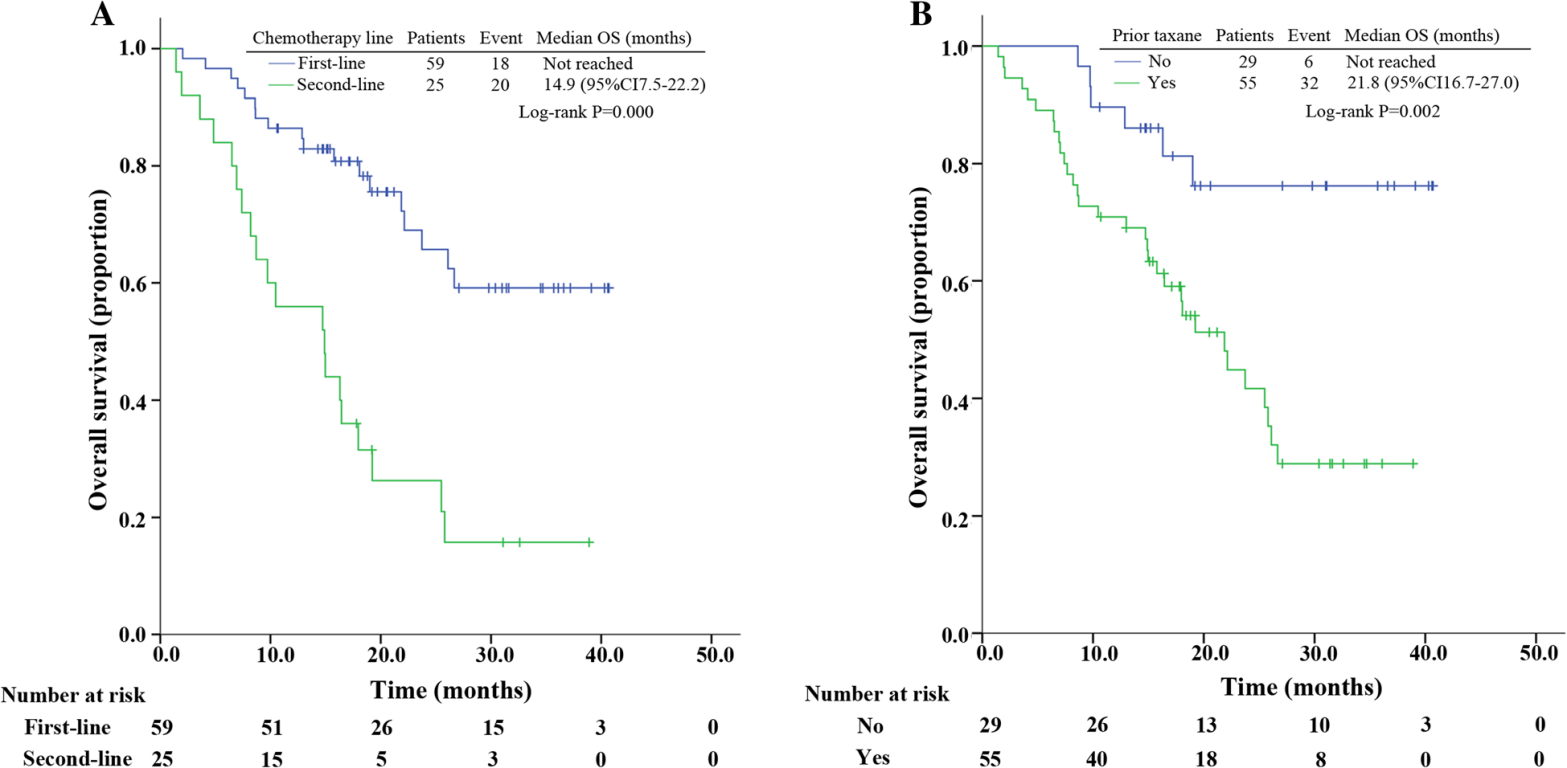
Kaplan–Meier curves for overall survival. **a** For patients stratified by lines of therapy. **b** For patients stratified by prior taxane. Abbreviations: CI, confidence interval; OS, overall survival

The median follow-up was 17.2 months. Two patients were lost during follow-up with no survival data. Median PFS was 7.9 months (95% CI, 6.6 to 9.2), while median OS was 25.8 months (95% CI, 20.4 to 31.1). In multivariate analyses, the median PFS was significantly longer in patients who received nab-paclitaxel and gemcitabine as first-line chemotherapy, as compared with those who received the combination as second-line treatment or more (hazard ratio [HR], 2.2; 95% CI 1.3 to 3.6) (Fig. 2a). The median OS was significantly longer in first-line patients than second-line or more (first-line, not reached; second-line or more, 14.9 months; Log-rank *p* = 0.000) (Fig. 3a). Median PFS of patients with or without prior taxanes was 10.7 and 6.9 months, respectively, with HR of 1.9 (95% CI 1.1–3.1) (Fig. 2b). Prior taxane was also identified as significant in OS (*p* = 0.002) (Fig. 3b). Longer PFS and OS were observed in patients who reached ORR, compared to patients who didn’t (Table 2). No significant difference was seen in PFS and OS of different molecular subtype (Table 2). Patients without liver metastasis had a significantly longer OS (*p* = 0.028), but a numerically longer PFS (*p* = 0.062), compared to patients with liver metastasis (Table 2).

**Table 2:** Treatment efficacy and subgroup analysis

Variable	N	Overall response rate (%)	*P*-value	Median progression-free survival (months) [95%CI]	Log-rank *P*-value	Hazard ratio for PFS [95% confidence interval]	Median overall survival (months) [95%CI]	Log-rank *P*-value	Hazard ratio for OS [95% confidence interval]
Whole group	84	52.4		7.9[6.6–9.2]			25.8[20.4–31.1]		
Lines of chemotherapy			0.044*		0.001*	2.2[1.3-3.6]		0.000*	3.8[2.0-7.2]
First-line	59	65.4		9.4[6.5–12.3]			Not reached		
Second-line or more	25	38.0		5.7[4.3–7.2]			14.9[7.5–22.2]		
Molecular subtypes			0.144		0.102	*n.s.*		0.08	*n.s.*
Luminal	61	54.4		8.2[6.8–9.5]			22.1[14.7–29.5]		
HER2 positive	10	87.5		10.5[7.3–13.5]			Not reached		
Triple-negative	12	50.0		5.1[4.3–5.9]			15.7[2.5–29.0]		
Prior taxane			0.429		0.007*	1.9[1.1-3.1]		0.002*	*n.s.*
Yes	55	47.3		6.9[5.1–8.6]			21.8[16.7–27.0]		
No	29	62.0		10.7[10.1–11.3]			Not reached		
Liver metastasis			0.017*		0.062	*n.s.*		0.028*	3.7[1.5-8.8]*.*
Yes	48	46.7		6.8[5.4–8.1]			21.8[16.9–27.1]		
No	36	74.2		8.6[5.6–11.6]			Not reached		
Overall response			–		0.000*	*n.s.*		0.007*	*n.s.*
ORR	44	–		10.4[8.0–12.8]			Not reached		
Non-ORR	32	–		4.8[2.8–6.8]			21.8[14.9–28.8]		

* *P*<0.05,statistical significance

The total number of delivered cycles in all patients was 360, with a median treatment course of 5 cycles (range: 1–8). Median duration of response was 6.1 months (95%CI 3.9 to 8.2). Nine patients had dose reductions: two had dose reduction of both drugs due to grade 3/4 febrile neutropenia; one due to concurrent pyothorax. Four patients reduced the dose of nab-paclitaxel due to grade 3 neuropathy, and one due to intolerable edema. One patient stopped gemcitabine due to interstitial pneumonia. The reasons for discontinuation are shown in Fig. 1.

### Safety

Patients who received at least one dose of nab-paclitaxel and gemcitabine were analyzed for safety. The toxicity profiles are summarized in Table 3. The most common grade 3/4 hematologic toxicity was neutropenia (45.24%), while only 2 patients developed febrile neutropenia (2.38%). The most common (> 10%) non-hematologic toxicities included neuropathy (50.00%), alopecia (40.48%), rash (33.33%), nausea and vomiting (25.00%), myalgia (23.80%), edema (23.80%), nail change (22.62%), and fatigue (16.67%).

**Table 3:** Adverse events

AE	All grade	3/4	1	2	3	4
Anemia	21(25.00%)	4(4.76%)	7(8.33%)	10(11.90%)	4(4.76%)	0
Neutropenia	63(75.00%)	38(45.24%)	7(8.33%)	18(21.43%)	21(25.00%)	17(20.24%)
Thrombocytopenia	17(20.24%)	7(8.33%)	1(1.19%)	9(10.71%)	6(7.14%)	1(1.19%)
Neuropathy	42(50.00%)	6(7.14%)	27(32.14%)	9(10.71%)	6(7.14%)	0
Diarrhea	2(2.38%)	0	1(1.19%)	1(1.19%)	0	0
Anorexia	3(3.57%)	0	3(3.57%)	0	0	0
Fatigue	14(16.67%)	0	9(10.71%)	5(5.95%)	0	0
Rash	28(33.33%)	2(2.38%)	15(17.86%)	11(13.09%)	2(2.38%)	0
Blurred vision	8(9.53%)	0	7(8.33%)	1(1.19%)	0	0
Dry eye	1(1.19%)	0	1(1.19%)	0	0	0
Eye pain	1(1.19%)	0	1(1.19%)	0	0	0
Alopecia	34(40.48%)	0	6(7.14%)	28(33.33%)	0	0
Nausea and vomiting	21(25.00%)	0	17(20.24%)	4(4.76%)	0	0
Nail change	19(22.62%)	0	15(17.86%)	4(4.76%)	0	0
Mucositis	6(7.14%)	0	2(2.38%)	3(3.57%)	1(1.19%)	0
Edema	20(23.80%)	0	14(16.67%)	6(7.14%)	0	0
Liver enzyme escalation	6(7.14%)	0	3(3.57%)	3(3.57%)	0	0
Hematuria	2(2.38%)	0	2(2.38%)	0	0	0
Skin hyperpigmentation	8(9.53%)	0	8(9.53%)	0	0	0
Hyperhidrosis	1(1.19%)	1(1.19%)	0	0	1(1.19%)	0
Febrile neutropenia	2(2.38%)	2(2.38%)	0	0	1(1.19%)	1(1.19%)
Myalgia	20(23.81%)	0	17(20.24%)	2(2.38%)	1(1.19%)	0
Hypersensitivity	1(1.19%)	1(1.19%)	0	0	1(1.19%)	0
Epistaxis	2(2.38%)	0	2(2.38%)	0	0	0
Abdominal distension	3(3.57%)	0	2(2.38%)	1(1.19%)	0	0
Pneumonitis	1(1.19%)	0	0	1(1.19%)	0	0

Grade 3 peripheral neuropathy, cumulative dose-limiting toxicity, occurred in six patients (7.1%), two of whom had nab-paclitaxel discontinued due to no recovery after a 2 weeks’ delay. 4 patients recovered to grade 2 or less neuropathy after delay and experienced dose reduction of nab-paclitaxel in subsequent courses.

Skin rash, another common toxicity, was observed in 29 patients (35.5%). Only three patients developed grade 3 skin toxicity with generalized erythroderma and disfigurement of the face. However, they recovered in time and no dose-reduction was needed.

### Tumor/stromal Cav-1 expression related to efficacy

**Fig. 4:**
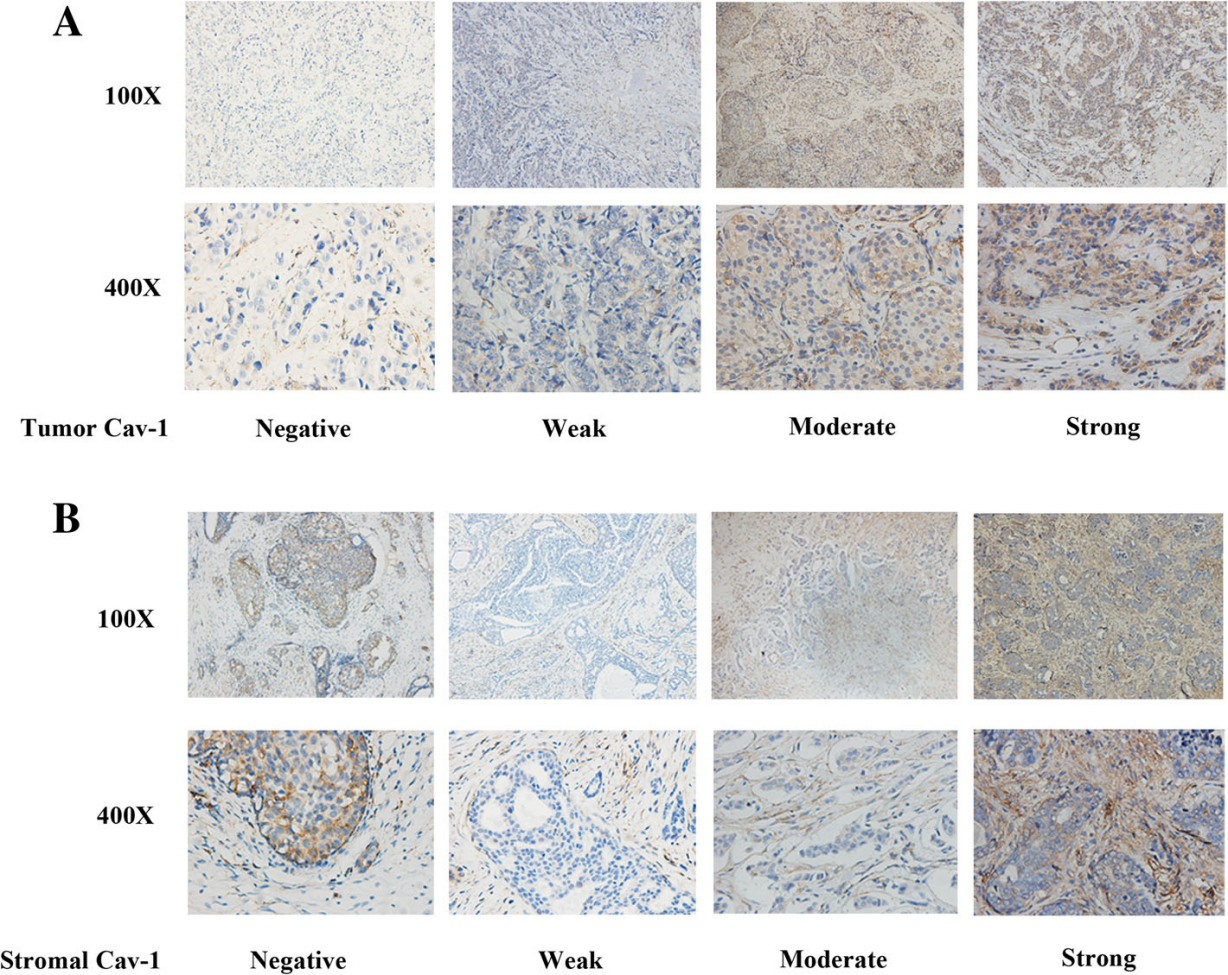
Representative immunohistochemistry images for patients with tumor/stromal Cav-1 staining. **a** Tumor exhibited strong intensity (3), moderate intensity (2), weak intensity (1) and negative (0) for Cav-1 (from right panel to left panel). **b** Stromal exhibited strong intensity (3), moderate intensity (2), weak intensity (1) and negative (0) for Cav-1 (from right panel to left panel). Abbreviations: Cav-1, Caveolin-1

FFPE tissue from primary breast cancer was requested from all enrolled patients, and 45 patients were available for FFPE tissue. We assessed Cav-1 expression of breast cancer tissue and stromal tissue through immunohistochemistry and set the cut-off point as median value of scores (Fig. 4). 27 patients were assessed as low expression and 18 as high expression in their breast cancer cells; 24 patients had low stromal Cav-1 expression and 21 as high stromal expression.

**Fig. 5:**
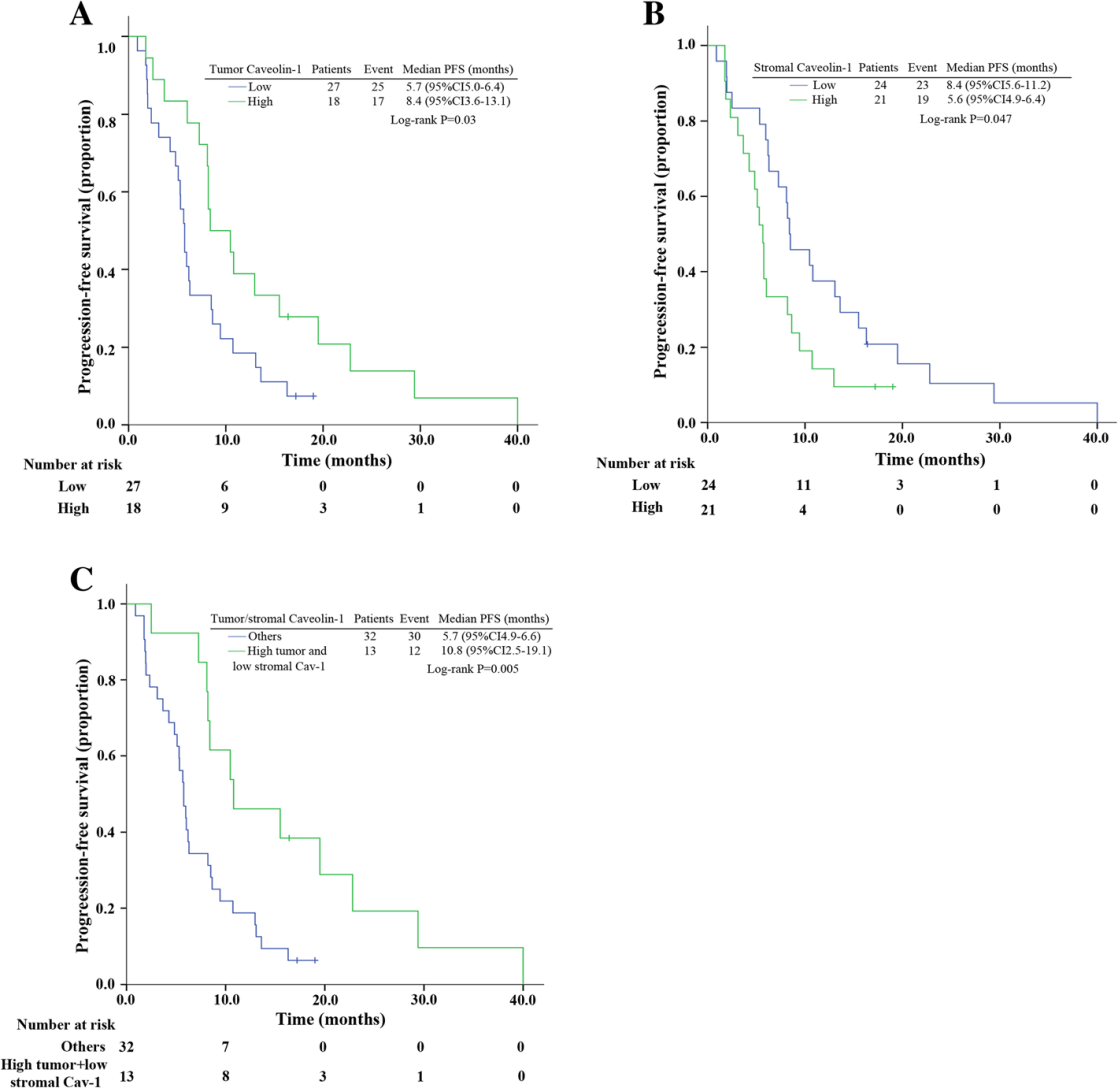
Kaplan–Meier curves for progression-free survival in patients with tumor/stromal Cav-1 staining. **a** For patients stratified by tumor Cav-1 staining. **b** For patients stratified by stromal Cav-1 staining. **c** For patients stratified by tumor and stromal Cav-1 staining. Abbreviations: CI, confidence interval; PFS, progression-free survival

Patient characteristics were balanced between low and high expression groups (Additional file 1: Table S1**)** except molecular subtypes. More patients with HER2+ and triple-negative breast cancer had low tumor Cav-1 expression (Chi-square test *p* = 0.006). For Cav-1 level in tumor and stromal tissue, they were not significantly associated with ORR (Additional file 2: Table S2). However, higher tumor Cav-1 level was significantly related with longer PFS of nab-paclitaxel and gemcitabine (Log-rank *p* = 0.03, Fig. 5a). For Cav-1 level in stromal tissue, significant longer PFS was noted in patients with lower Cav-1 level (Log-rank *p* = 0.047, Fig. 5b). Patients with high tumor but low stromal Cav-1 staining (13/45) had the longest PFS, with a median PFS of 10.1 months (95% CI 2.4–19.1, Fig. 5c). No significant association was observed between OS and tumor/stromal Cav-1 expression (Additional file 3: Figure S2).

## Discussion

The study herein presented was phase II, single arm, prospective study, originally conducted to evaluate the efficacy and safety of weekly nab-paclitaxel plus gemcitabine therapy in pretreated Chinese patients with MBC. The obtained results showed that the aforementioned combination had a substantial antitumor activity with a tolerable safety profile. ORR of 52.4% (CR 2.4% and PR 50%) was observed in FAS patients, with median PFS of 7.9 months (95% CI 6.6–9.2) and median OS of 25.8 months (95% CI 20.4–31.1). The regimen was well tolerated. The incidence of grade 3/4 neutropenia was 45.24%. Only six patients (7.14%) experienced grade 3 neurotoxicity, which led to dose reduction or discontinuation. Hypersensitivity reactions were observed in only one patient who received no routine premedication of paclitaxel.

Compared with the existing studies of nab-paclitaxel and gemcitabine combination, our study revealed a better efficacy and safety profile (Table 4). Roy et al. have administered nab-paclitaxel and gemcitabine as the first-line treatment for MBC patients and gained an ORR of 50%, and median PFS of 7.9 months [6]. As the first-line treatment, our study showed superior results compared with the existing ones (mPFS: 9.4 vs.7.9 months,ORR:65.4% vs. 50%). This difference in efficacy might be due to different chemotherapy schedule. In Roy’s study, fifty patients have received a treatment with nab-paclitaxel (125 mg/m^2^) and gemcitabine (1000 mg/m^2^) on day 1 and 8 of a 21-day cycle. Whereas, we administered weekly nab-paclitaxel and gemcitabine in a three weeks on and one week off cycle and a less dose of gemcitabine (800 mg/m^2^) every week. The standard dose intensity of nab-paclitaxel was 93.7 mg per square meter per week in our study, compared to 83.3 mg per square meter per week in Roy’s study. With reference to safety profile, Roy et al. have reported that incidence of grade 3/4 neutropenia was 54% compared with ours 45.2%, while the incidence of grade 3/4 neuropathy was 8%, which was similar with ours at 7–8%. Our study showed that combination of nab-paclitaxel and gemcitabine administered on day 1, 8, and 15 in a 4-week cycle may be associated with improved tolerability compared to a day 1 and 8 in a 3-week cycle.

**Table 4:** Nab-paclitaxel-containing regimens for metastatic breast cancer

Study	Regimen	Study design and setting	Number of patients	Overall response rate	Median time to progression/progression-free survival (months)	Median overall survival (months)
Our study	Nab-paclitaxel 125 mg/m^2^+ GEM 800 mg/m^2^, days 1, 8, 15, q4w	Phase II,all lines	84	52.40%	7.9	25.8
Gradishar et al. [3]	Nab-paclitaxel 260 mg/m^2^,q3w	Phase III,all lines	229	33%	23.0(weeks)	NR
	Paclitaxel 175 mg/m2,q3w		225	19%	16.9(weeks)	NR
Gradishar et al. [14]	Nab-paclitaxel 300 mg/m^2^,q3w	Phase II, first-line	76	37%	11	27.7
	Nab-paclitaxel 100 mg/m^2^ weekly, days 1, 8, 15, q4w		76	45%	12.8	22.2
	Nab-paclitaxel 150 mg/m^2^ weekly, days 1, 8, 15, q4w		74	49%	12.9	33.8
	Docetaxel 100 mg/m^2^,q3w		74	35%	7.5	26.6
Roy et al. [6]	Nab-paclitaxel 125 mg/m^2^ weekly, days 1,8,q3w + GEM 1000 mg/m^2^ weekly, days 1,8,q3w	Phase II, first-line	50	50%	7.9	NR
Lobo et al. [7]	Nab-paclitaxel 150 mg/m2 + GEM 1500 mg/m2 + bevacizumab 10 mg/kg,day 1,15,q4w	Phase II, first-line, TNBC	30	75.90%	10.4	NR
Sun et al. [13]	Nab-paclitaxel 125 mg/m2, days 1, 8, 15+ DDP 75 mg/m2, q4w	Phase II,all lines	73	67.10%	9.8	26.9

Abbreviations: q3w: every 3 weeks, GEM: gemcitabine, DDP: cisplatin, TNBC: tri-negative breast cancer

Concerning subgroup analysis, 65.5% of patients had previously received a taxane in adjuvant therapy and/or in metastatic therapy. There was significant difference in PFS and OS based on prior taxane administration, which has not been reported by Roy et al [6] Though the activity of paclitaxel was enhanced when bound with albumin and delivered by a serial of albumin receptors (including gp60 and Cav-1), patients previously treated with taxane showed to be less sensitive when exposed to nab-paclitaxel. And taxane-naive MBC patients had a PFS of 10.7 months, which was longer than combination of GT (mPFS 6 months) [5] or capecitabine and docetaxel (mTTP 6.1 months) [11].

Even though there is no phase III trial that directly compares weekly nab-paclitaxel and weekly paclitaxel monotherapy in metastatic breast cancer patients, weekly nab-paclitaxel showed to have a favorable anti-tumor activity compared to weekly paclitaxel. On the one hand, GeparSepto-GBG 69 trial has showed that in neoadjuvant setting, weekly nab-paclitaxel could significantly increase the proportion of patients achieving pathological complete response (pCR) compared with weekly solvent-based paclitaxel [12]. Furthermore, among different subtypes, triple-negative breast cancer reached the highest effect from weekly nab-paclitaxel with an odds ratio of 2.61 (95%CI 1·57–4·33). As neoadjuvant therapy is an effective way to test novel agents and offer a potentially rapid and efficient strategy for drug development, we believe that weekly nab-paclitaxel has an improved efficacy compared to weekly paclitaxel. On the other hand, in our previous study, we identified a highly effective doublet of nab-paclitaxel and cisplatin in different lines of metastatic breast cancer patients. An enhanced efficacy was observed with ORR of 67.1%, mPFS of 9.8 months and OS of 26.9 months in 73 enrolled patients [13]. These findings can serve as a powerful evidence to show the extraordinary antitumor effects of weekly nab-paclitaxel. However, a higher incidence of hematologic toxicity (grade 4 neutropenia 63.0%), febrile neutropenia (12.3%) and peripheral neuropathy (grade 3 neuropathy 26%) was also observed, compared with nab-paclitaxel and gemcitabine combination. Subsequently, we substituted cisplatin with gemcitabine in order to reach a high efficacy with a more tolerable toxicity.

The present study furthermore revealed that nab-paclitaxel plus gemcitabine therapy was well tolerated in Chinese patients, with a median course of 5 cycles. The most common adverse events encountered following weekly nab-paclitaxel alone were neutropenia (44%) and sensory neuropathy (14%) [14]. The incidence of adverse events did not increase with the combination of gemcitabine. Paclitaxel and gemcitabine could potentially contribute to neurotoxicity, but they didn’t have a synergistic adverse effect when combined. Gradishar et al. have reported that the median recovery time to grade 2 or less for nab-paclitaxel-induced neurotoxicity is about 22 days. After 2 weeks of recovery, 78.6% of grade 3 patients still do not recover to grade 2 or less [3]. The addition of gemcitabine did not intensely increase the incidence of grade 3/4 neuropathy or the number of grade 3 neuropathy patients with prolonged recovery time. Existing studies have revealed that the incidence of rash reported in Chinese patients treated with nab-paclitaxel-containing combination is 37.0% (grade 3, 1.37%) [15]. We observed an all-grade incidence of rash 33.33% (grade 3, 2.38%), showing a higher rate in Chinese patients compared to Westerner (26.9%).

This study is the first to demonstrate a role for tumor/stromal Cav-1 in breast cancer as a predictive biomarker for efficacy outcomes of nab-paclitaxel and gemcitabine. Higher tumor Cav-1 levels and lower stromal Cav-1 levels were significantly associated with longer PFS of nab-paclitaxel and gemcitabine. It is controversial about the distribution of Cav-1 in normal and invasive breast cancer in recent studies. Decreased tumor Cav-1 mRNA level is associated with low ER and PR and high HER2 expression in breast cancer, which is consistent with our study [16]. However, other studies indicate that high Cav-1 expression is associated with basal-like breast cancer and has positive correlation with high histological grade, lack of ER and PR, and expression of basal markers (basal cytokeratins, P63, P-cadherin), which reported a different result with our study [17, 18]. The reason for strong association between Cav-1 expression and basal-like breast cancer is the preference of Cav-1 expression in myoepithelial/basal cells of normal breast, which is the origin of basal-like breast cancer. While the majority of triple-negative breast cancers are basal-like, only 4 triple-negative breast cancers were included in Cav-1 staining analysis in our study and we didn’t test the expression of basal markers concurrently in these patients. Maybe these 4 breast cancers were not basal-like subtype or the difference of Cav-1 expression between subtypes mainly came from HER2-positive breast cancer. We will detect the expression of Cav-1 with a bigger sample size in further study to explain the difference between subtypes. Though subtypes of breast cancer were not well balanced in patients with different tumor Cav-1 expression, the doublet seemed to be equally effective across these molecular subtypes as shown above. Therefore, different subtypes might not influence the prediction of tumor Cav-1 expression on PFS.

The reason that tumor Cav-1 levels serve as a predictive biomarker is mainly the role of Cav-1 in the transport of nab-paclitaxel. The prominent transport way of nab-paclitaxel is acting through albumin-mediated transcytosis [8], while Cav-1 mediates transport of albumin through a gp60-dependent pathway [19, 20]. A preclinical study shows that Cav-1 expression mediates albumin uptake in cancer cells and directly determines the uptake of nab-paclitaxel in cancer cells [21]. Cav-1 overexpression enhances uptake and sensitivity to nab-paclitaxel and decreased Cav-1 conferred resistance in cancer cells and in xenograft models. We suppose that breast cancer patients with higher tumor Cav-1 expression may transport more nab-paclitaxel into the breast cancer cells through transcytosis and show better efficacy with higher tumor intracellular concentrations of nab-paclitaxel. Decreased stromal Cav-1 levels did significantly correlate with longer PFS of nab-paclitaxel and gemcitabine. One possible explanation is that high stromal Cav-1 levels allow for increased nab-paclitaxel uptake in stromal cells and tissue, which competitively reduce the uptake of nab-paclitaxel in the breast cancer cells. Patients with high stromal Cav-1 levels had a worse efficacy outcome for nab-paclitaxel and gemcitabine. Patients with high tumor but low stromal Cav-1 staining reached a longest PFS, which suggests that an optimal intratumor drug distribution is more drugs in tumor cells and less in stromal cells. Conversely, downregulation of Cav-1 expression in stroma plays a tumor-promoting role and predicts early cancer recurrence, lymph node infiltration, and chemotherapeutic resistance almost in all subtypes of breast cancer [10, 17, 22]. Even though, the efficacy of nab-paclitaxel predicted by stromal Cav-1 levels seems not to be influenced by the tumor-promoting functions of Cav-1 in stromal tissue. Regardless, additional validation of tumor/stromal Cav-1 in breast cancer patients as a predictive biomarker for nab-paclitaxel therapy is warranted, and tumor/stromal Cav-1 of metastatic sites through re-biopsy may better reflect the efficacy of nab-paclitaxel in metastatic breast cancer patients.

We have to point out that our study has some limitations. Firstly, our study is a single-arm trial with no control group. We compared the obtained results with the previous studies, which mean that the baseline characteristics may lack equivalency. This in turn may reduce the power of our hypothesis. Secondly, due to the nature of the study and restricted funds, we had a limited sample size. Thirdly, follow-up time was insufficient to obtain more convincing OS data.

## Conclusions

Nab–paclitaxel and gemcitabine combination is an effective and well-tolerated chemotherapy regimen for patients with MBC. Higher tumor Cav-1 levels and lower stromal Cav-1 levels were significantly associated with longer PFS of nab-paclitaxel and gemcitabine. The promising results of this trial present a strong rationale for a future phase III trial with the comparison of nab–paclitaxel+gemcitabine and paclitaxel+gemcitabine.

## Additional files

Additional file 1: **Table S1.** Characteristics for patients with tumor/stromal staining. (DOCX 18 kb)

Additional file 2: **Table S2.** Comparison of objective response in patients with different tumor/stromal Cav-1 expression. (DOCX 16 kb)

Additional file 3: **Figure S1.** Kaplan–Meier curves for overall survival in patients with tumor/stromal Cav-1 staining. (A) For patients stratified by tumor Cav-1 staining. (B) For patients stratified by stromal Cav-1 staining. (C) For patients stratified by tumor and stromal Cav-1 staining. Abbreviations: CI, confidence interval; PFS, progression-free survival. (TIF 1011 kb)

## Abbreviations

Cav-1: Caveolin-1
CR: Complete response
ECOG: Eastern Cooperative Oncology Group
FFPE: Formalin fixed paraffin-embedded
MBC: Metastatic breast cancer
nab-paclitaxel: Nanoparticle albumin-bound paclitaxel
NCCN: National Comprehensive Cancer Network
NCI-CTCAE: National Cancer Institute Common Terminology Criteria for Adverse Events
ORR: Objective response rate
OS: Overall survival
pCR: Pathological complete response
PD: Progression disease HR Hazard ratio
PFS: Progression free survival
PR: Partial response
RECIST: Response Evaluation Criteria in Solid Tumors
SD: Stable disease
TNBC: Triple-negative breast cancer
