# Supplementary material for: Caveolin-1 expression predicts efficacy of weekly nab-paclitaxel plus gemcitabine for metastatic breast cancer in the phase II clinical trial
Source: BMC Cancer. 2018 Oct 22;18:1019. doi: 10.1186/s12885-018-4936-y (PMC6196471; doi:10.1186/s12885-018-4936-y)
Supplement: Supplementary file 1 — Table S1. Characteristics for patients with tumor/stromal staining. (DOCX 18 kb) [file 12885_2018_4936_MOESM1_ESM.docx]

**Supplementary Table1. Characteristics for patients with tumor/stromal staining**

| **Characteristic** | **Number** | | | | | |
| --- | --- | --- | --- | --- | --- | --- |
|  | **Tumor Cav-1** | | | **Stromal Cav-1** | | |
|  | **Low** | **High** | **P value** | **Low** | **High** | **P value** |
| **No. of metastatic sites** |  |  |  |  |  |  |
| 1 | 3 | 0 | 0.15 | 2 | 1 | 0.24 |
| 2 | 9 | 9 |  | 12 | 6 |  |
| ≥3 | 15 | 9 |  | 10 | 14 |  |
| **Metastatic sites** |  |  |  |  |  |  |
| Visceral | 22 | 15 | 1.00 | 18 | 19 | 0.34 |
| Non-visceral | 5 | 3 |  | 6 | 2 |  |
| Liver | 15 | 11 | 0.95 | 12 | 14 | 0.41 |
| **Subtype** |  |  |  |  |  |  |
| Luminal | 18 | 17 | 0.006^*^ | 19 | 16 | 0.022^*^ |
| Triple-negative | 4 | 0 |  | 0 | 4 |  |
| Her-2 positive | 5 | 0 |  | 4 | 1 |  |
| Unknown | 0 | 1 |  | 1 | 0 |  |
| **Lines of chemotherapy** |  |  |  |  |  |  |
| First line | 15 | 13 | 0.42 | 15 | 13 | 1.00 |
| Second line or more line | 12 | 5 |  | 9 | 8 |  |
| **Prior taxane** |  |  |  |  |  |  |
| Yes | 21 | 14 | 1.00 | 7 | 3 | 0.40 |
| No | 6 | 4 |  | 17 | 18 |  |
