# Supplementary material for: Caveolin-1 expression predicts efficacy of weekly nab-paclitaxel plus gemcitabine for metastatic breast cancer in the phase II clinical trial
Source: BMC Cancer. 2018 Oct 22;18:1019. doi: 10.1186/s12885-018-4936-y (PMC6196471; doi:10.1186/s12885-018-4936-y)
Supplement: Supplementary file 2 — Table S2. Comparison of objective response in patients with different tumor/stromal Cav-1 expression. (DOCX 16 kb) [file 12885_2018_4936_MOESM2_ESM.docx]

**Supplementary Table2. Comparison of objective response in patients with different tumor/stromal Cav-1 expression**

| **Objective response** | **Number** | | | | | |
| --- | --- | --- | --- | --- | --- | --- |
|  | **Tumor Cav-1** | | | **Stromal Cav-1** | | |
|  | **Low** | **High** | **P value** | **Low** | **High** | **P value** |
| CR | 0 | 2 |  | 2 | 0 |  |
| PR | 11 | 9 |  | 10 | 10 |  |
| SD | 8 | 5 |  | 6 | 7 |  |
| PD | 6 | 1 |  | 3 | 4 |  |
| Unconfirmed | 2 | 1 |  | 3 | 0 |  |
| ORR |  |  | 0.405 |  |  | 0.283 |
