# Supplementary figures and images for: Caveolin-1 expression predicts efficacy of weekly nab-paclitaxel plus gemcitabine for metastatic breast cancer in the phase II clinical trial
Source: BMC Cancer. 2018 Oct 22;18:1019. doi: 10.1186/s12885-018-4936-y (PMC6196471; doi:10.1186/s12885-018-4936-y)

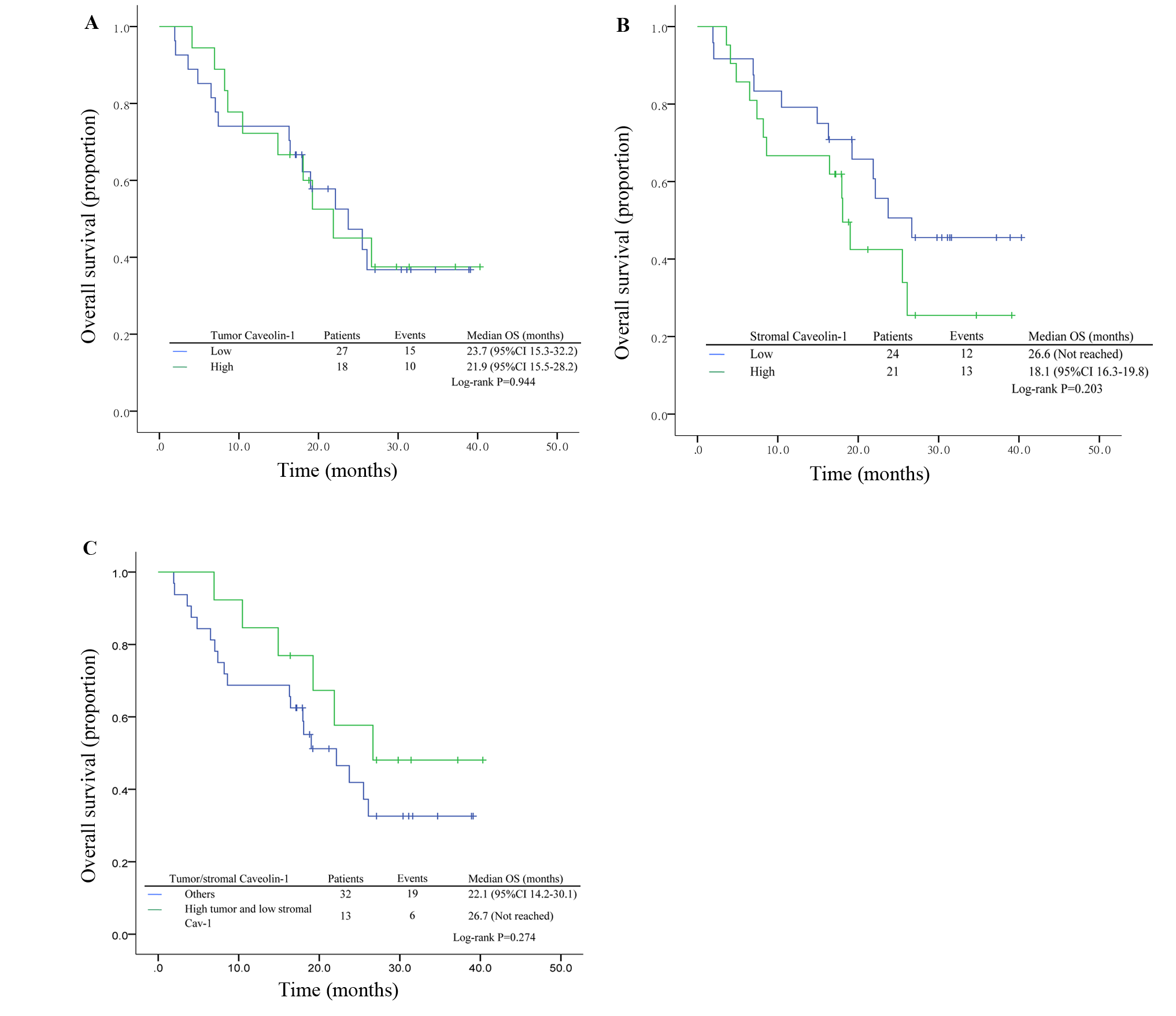

Supplement: Supplementary file 3 — Figure S1. Kaplan–Meier curves for overall survival in patients with tumor/stromal Cav-1 staining. (A) For patients stratified by tumor Cav-1 staining. (B) For patients stratified by stromal Cav-1 staining. (C) For patients stratified by tumor and stromal Cav-1 staining. Abbreviations: CI, confidence interval; PFS, progression-free survival. (TIF 1011 kb) [file 12885_2018_4936_MOESM3_ESM.tif]
